# Transcriptomics reveal different metabolic strategies for acid resistance and gamma-aminobutyric acid (GABA) production in select *Levilactobacillus brevis* strains

**DOI:** 10.1186/s12934-021-01658-4

**Published:** 2021-09-06

**Authors:** Sagarika Banerjee, Matthew Poore, Svetlana Gerdes, Derek Nedveck, Lene Lauridsen, Heidi Thomsen Kristensen, Henrik Max Jensen, Phillip M. Byrd, Arthur C. Ouwehand, Elaine Patterson, Wesley Morovic

**Affiliations:** 1 IFF Health and Biosciences, Danisco USA, Inc., Madison, WI USA; 2 IFF Health and Biosciences, Brabrand, Denmark; 3 IFF Health and Biosciences, Danisco Sweeteners Oy, Sokeritehtaantie 20, 02460 Kantvik, Finland

**Keywords:** Gamma-aminobutyric acid, GABA, *Levilactobacillus brevis*, Transcriptomics, Acid resistance, Comparative genomics, Glutamate, Gut microbiota, Gut-brain axis, Stress, Mental health

## Abstract

**Background:**

Of the many neurotransmitters in humans, gamma-aminobutyric acid (GABA) shows potential for improving several mental health indications such as stress and anxiety. The microbiota-gut-brain axis is an important pathway for GABAergic effects, as microbially-secreted GABA within the gut can affect host mental health outcomes. Understanding the molecular characteristics of GABA production by microbes within the gut can offer insight to novel therapies for mental health.

**Results:**

Three strains of *Levilactobacillus brevis* with syntenous glutamate decarboxylase (GAD) operons were evaluated for overall growth, glutamate utilization, and GABA production in typical synthetic growth media supplemented with monosodium glutamate (MSG). *Levilactobacillus brevis* Lbr-6108™ (Lbr-6108), formerly known as *L. brevis* DPC 6108, and *Levilactobacillus brevis* Lbr-35 ™ (Lbr-35) had similar growth profiles but differed significantly in GABA secretion and acid resistance. Lbr-6108 produced GABA early within the growth phase and produced significantly more GABA than Lbr-35 and the type strain *Levilactobacillus brevis* ATCC 14869 after the stationary phase. The global gene expression during GABA production at several timepoints was determined by RNA sequencing. The GAD operon, responsible for GABA production and secretion, activated in Lbr-6108 after only 6 h of fermentation and continued throughout the stationary phase. Furthermore, Lbr-6108 activated many different acid resistance mechanisms concurrently, which contribute to acid tolerance and energy production. In contrast, Lbr-35, which has a genetically similar GAD operon, including two copies of the GAD gene, showed no upregulation of the GAD operon, even when cultured with MSG.

**Conclusions:**

This study is the first to evaluate whole transcriptome changes in *Levilactobacillus brevis* during GABA production in different growth phases. The concurrent expression of multiple acid-resistance mechanisms reveals niche-specific metabolic functionality between common human commensals and highlights the complex regulation of GABA metabolism in this important microbial species. Furthermore, the increased and rapid GABA production of Lbr-6108 highlights the strain’s potential as a therapeutic and the overall value of screening microbes for effector molecule output.

**Supplementary Information:**

The online version contains supplementary material available at 10.1186/s12934-021-01658-4.

## Background

In lactic acid bacteria (LAB), gamma-aminobutyric acid (GABA) is produced by glutamate decarboxylation encoded by the glutamate decarboxylase or glutamic acid decarboxylase (GAD) operon. Extracellular glutamate may also be shunted away from the GAD pathway into the tricarboxylic acid cycle for charging tRNA by the action on glutamyl-tRNA synthetase or synthesized de novo from glucose in the glycolysis pathway [1]. The GAD operon consists of three important elements responsible for GABA secretion in bacteria: the positive transcriptional regulator encoded by *gadR* gene, the glutamate/GABA antiporter encoded by *gadC* gene, and the glutamate decarboxylase enzyme encoded either by *gadA* or *gadB* genes [2, 3]. The number of *gadA* or *gadB* copies in different LAB genera like *Enterococcus, Streptococcus, Lactococcus, Pediococcus, Propionibacterium,* and *Lactobacillus* (sensu lato) are variable [1]. Interestingly, while most GAD systems in LAB have one GABA producing *gad* (*A* or *B*) gene, *Levilactobacillus brevis* is the only known species that encodes two biochemically identical isoforms of the GAD enzyme [3, 4]. However, the genetic organization of the GAD system could also be different between strains of *L. brevis,* with most strains having two copies of the GAD encoding genes [1]. In the *L. brevis* chromosome, the GAD operon includes *gadC* and *gadB* genes, while the *gadA* gene is far downstream from the *gad* operon [3]. The GAD operon also includes *gadR*, which positively regulates GABA production in a glutamate-dependent manner and is essential for GABA conversion from glutamate [5].

GABA is the main inhibitory neurotransmitter in the body and central nervous system (CNS), functioning to reduce the activity of the neurons to which it binds, inhibiting nerve transmission and counterbalancing the action of the excitatory neurotransmitter glutamate [6–8]. The effects of GABA are mediated through ionotropic GABA_A_ receptors, which exist as a number of subtypes formed by the co-assembly of different subunits (α, β, and γ subunits) and metabotropic GABA_B_ receptors, which are G protein-coupled receptors, which consist of a heterodimer made up of two subunits (GABA_B1_ and GABA_B2_), whereby both are necessary for receptor functionality [9, 10]. Clinically relevant pharmaceuticals such as anxiolytics and skeletal muscle relaxants target GABA receptors (e.g., benzodiazepines acting on GABA_A_ receptors and baclofen acting on GABA_B_ receptors). Thus, alterations in GABAergic neurotransmission has important roles in the development of stress-related psychiatric conditions. GABA receptors are also widely distributed throughout the body and complementary to the CNS function of GABA, the multifunctional role of GABA has been investigated in the enteric nervous system (ENS) located all along the gastrointestinal tract, regulating functions such as intestinal motility, gastric emptying, nociception, and acid secretion [11], in the pancreas and even in immune cells [12, 13]. GABA is a non-proteinogenic amino acid and exists naturally in various foods and beverages such as tea, tomato, soybean, germinated rice, and some fermented foods such as kimchi and fermented fish. More recently, GABA food supplements have become a popular way to naturally increase dietary GABA, with potential health benefits [14, 15]. Understanding how GABA affects mental health is hugely important since mental illness is predicted to have a global cost of $16 trillion by 2030 [16].

The microbiota-gut-brain axis is a bidirectional communication pathway between the CNS and the gut microbiota, which is mediated by several direct and indirect pathways within the gut-brain axis [17]. Previous research has shown that germ-free animals, those lacking any microbial exposure since birth, had reduced luminal and serum levels of GABA [18], indicating that microbes within the gut can produce host-recognized GABA in situ [19]. Further research has shown that commensal organisms, such as LAB, play a major role in gut-brain signaling [20–24]. For instance, supplementation with *Lacticaseibacillus rhamnosus* JB-1 was previously shown to reduce stress-induced corticosterone and anxiety- and depression-related behavior in mice, while also inducing region-dependent alterations in GABA receptor expression in the brain [25]. This seminal study highlighted the role of the gut microbiota in influencing signaling pathways along the microbiota-gut-brain axis to affect brain function and behavior.

The production of GABA using the GAD operon confers glutamate-dependent acid resistance with *gadA* also retaining its activity toward near-neutral cytosolic pH (pH 5.5–6.5) [3]. The function of the *gadC* antiporter is activated under acidic (pH 3.5–5.5) conditions [3, 26], mostly at stationary phase of growth when there is extracellular and intracellular acidification. Thus, in *L. brevis* the two GAD genes together can contribute to efficient GABA synthesis capability in a broad pH range (pH 3.0–6.6) [3, 4], making *L. brevis* the highest GABA producing species amongst LAB [4, 5, 27]. Additionally, LAB have evolved other acid resistance mechanisms including the F0F1-ATPase system, amino acid/cation:proton antiporters, tyrosine decarboxylase system, agmatine deiminase system, arginine deiminase system, and the urea system [3, 28]. These systems all counteract the acid stress produced during fermentation or encountered within the host.

A previous study to screen human intestine-derived bifidobacteria and lactobacilli for the ability to produce GABA from monosodium glutamate (MSG) revealed the highest GABA producing isolate to be *L. brevis* DPC 6108, now commercially known as *Levilactobacillus brevis* Lbr-6108™ (Lbr-6108), an isolate cultured from an infant fecal sample [29]. Lbr-6108 was previously shown to increase serum insulin levels in healthy rats [30]. Later, in a mouse model of diet-induced obesity, obese mice supplemented with Lbr-6108 for twelve weeks showed improvements to several metabolic abnormalities associated with metabolic dysfunction, reduced depressive-like behavior, and increased endogenous GABA concentrations in the small intestine [31]. Taken together, these studies indicate a potential role of microbially-produced GABA by Lbr-6108 to influence host health [31]. We hypothesized that Lbr-6108 may be genetically distinct and predominantly use the GAD system as a preferred acid tolerance mechanism and could therefore be a potent source of microbial-derived GABA for the host. In this paper, we investigated the GABA production and growth characteristics of Lbr-6108 through a comparison to two other *L. brevis* strains, the commercial probiotic *L. brevis* Lbr-35™ (Lbr-35) and the type strain *L. brevis* ATCC 14869 (ATCC 14869) using in vitro methods complemented with RNA sequencing to explore the unique GABA producing capabilities of Lbr-6108.

## Results

### Genomic analysis of *Levilactobacillus* Lbr-6108, Lbr-35 and ATCC 14869 shows GAD operon synteny.

**Fig. 1:**
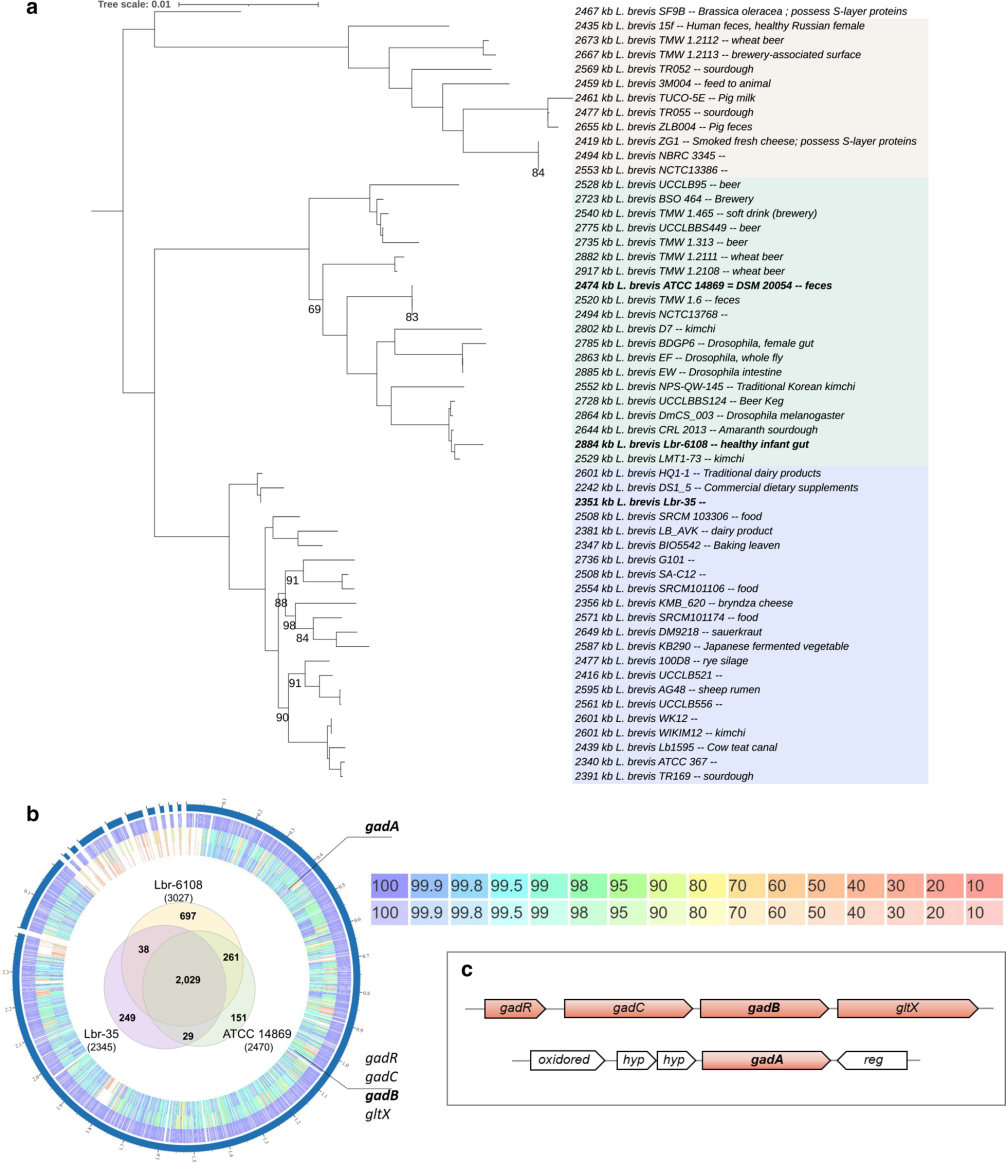
Genomic comparison of the GAD operon in *Levilactobacillus brevis* strains. **A** Phylogenetic tree of the *L. brevis* species based on 500 core proteins using RAxML in the Phylogenetic Tree Building Service of the PATRIC database [32]. Support values were generated using 100 rounds of the ‘Rapid’ bootstrapping option of RAxML; only bootstrap values lower than 100 are shown. Tree scale is given in amino acid substitutions per site. Genome sizes are shown to the left of each leaf. The three different clades are shaded different colors. **B** The circular genome map compares overall protein homology across the genome, with rings from the inside out representing ATCC 14869, Lbr-35, and Lbr-6108. The Venn diagram shows number of shared genes. The heat map shows percent protein sequence identity of the bidirectional best hit (top) and unidirection-al best hit (bottom). **C** An overview of the genes for glutamate metabolism in *L. brevis*, including the canonical GAD operon and the separate *gadA*

The total genome sizes of Lbr-6108 and ATCC 14869 are 2,884,677 bp and 2,473,148 bp, respectively. The novel draft genome of Lbr-35 is in six contiguous sequences with a total size of 2,338,793 bp and a G + C content of 46.1%. Further, the Lbr-35 genome annotation revealed 2,328 coding regions, 62 tRNAs, 15 rRNAs, and single CRISPR repeat-spacer array of 15 spacers. Phylogenomic analysis using all publicly available genomes revealed three major clades of *L. brevis* (Fig. 1A). Lbr-6108 and ATCC 14869 are both fecal isolates belonging to the same clade, while Lbr-35 is in a separate strain group. Lbr-35 is a commercial probiotic and the isolation source is not known, however, it groups most similarly to *L. brevis* HQ1-1, isolated from traditional dairy products. While the three *L. brevis* strains shared 2,029 genes, Lbr-6108 had 697 unique genes in an additional ~ 500,000 bp genomic region. Thirty-eight genes are shared between Lbr-6108 and Lbr-35, and 261 genes are shared between Lbr-6108 and ATCC 14869 (Fig. 1B). The larger genomic size of Lbr-6108 accounts for one or more large plasmids and many unique genes such as prophage associated proteins and genes of de novo fatty acid biosynthesis along with some regulators and genes for phosphotransferase uptake systems.

*L. brevis* has been shown to uniquely encode two GAD genes [1]. Indeed, all three *L. brevis* strains assessed here had both copies of *gadA* and *gadB*. The *gadB* gene is in the GAD operon together with transcriptional regulator (*gadR*), glutamate/gamma-aminobutyrate antiporter (*gadC*) and glutamyl-tRNA synthase (*gltX*), while the *gadA* gene is a stand-alone gene (Fig. 1C). Pairwise nucleotide alignment of the entire GAD operon shows Lbr-6108 to be 99.0% and 99.3% identical to Lbr-35 and ATCC 14869, respectively. No large insertions or deletions were found. While comparing amino acid sequences of the individual proteins, Lbr-6108 had 100.0% amino acid sequence identity to Lbr-35 for *gadR*, *gadC*, and *gadB*. The same comparison with ATCC 14869 showed single mutations in *gadR*, *gadC*, and *gadB* compared to Lbr-6108. The Lbr-6108 *gadA* protein sequence was 98.9% and 99.8% (refer PATRIC?) identical to that of Lbr-35 and ATCC 14869, respectively. Three promoter regions were predicted in the intergenic region directly upstream of *gadR* in Lbr-6108. The intergenic sequence for two of the three promotor regions in Lbr-35 is highly polymorphic, which may influence the overall expression of the GAD operon.

### Lbr-6108 rapidly produces GABA, starting early in fermentation.

**Fig. 2:**
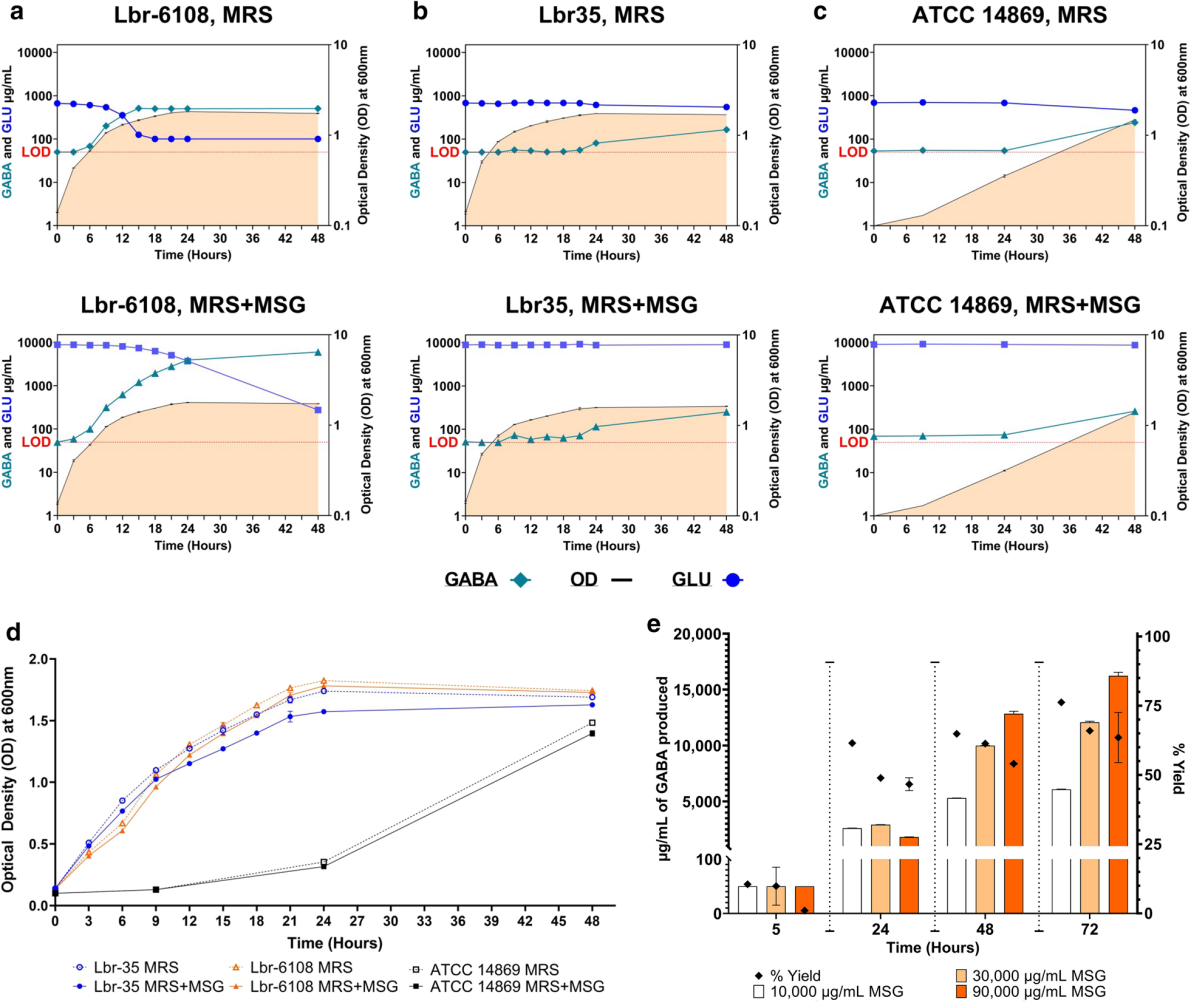
Microbial GABA production and glutamate utilization by *Levilactobacillus brevis* strains. GABA and glutamate are measured from cell free culture supernatants by liquid chromatography–mass spectrometry and are expressed in units of μg/mL. Limit of detection (LOD) for quantitation of GABA is 50 μg/mL and for glutamate is 100 μg/mL. Symbols are further explain un-der each graph type. **A**–**C** GABA (teal and l-glutamic acid (blue) measured in the culture supernatants (right y-axis) at each of the timepoints (x-axis) during growth (OD at 600 nm on the right hand y-axis) of the *L. brevis* strains Lbr-6108 (**B**), Lbr-35 (**C**) and ATCC 14869 (**D**) in MRS media without (dotted lines and open symbols) and with 10,000 μg/mL monosodium glutamate (MSG) (solid lines and closed symbols). **D** Growth curve of all *L. brevis* strains in MRS media with and without 10,000 μg/mL MSG for comparison. **E** GABA production over time when Lbr-6108 was grown in MRS media supplemented with 10,000 μg/mL, 30,000 μg/mL or 90,000 μg/mL of MSG. The bar graph represents GABA production (left y-axis) in µg/mL of GABA produced, while the black diamonds shows the percent of yield (calculated as GABA μg/mL/glutamate utilization μg/mL) (right y-axis). Error bars may be obscured by symbols and boxes

All three strains were grown in De Man-Rogosa-Sharpe broth (MRS) or MRS + MSG at an initial pH of media between 5.5 and 6. There was no significant difference between Lbr-6108 and Lbr-35 at T48 in either media, although ATCC 14869 was significantly lower than Lbr-6108 at T48 (Fig. 2A–C, Additional file 1: Table S1). The slower growth rate was not in proportion to the lower inoculation in ATCC 14869 (average of OD 0.040 lower) (Additional file 1: Table S1). While the growth of Lbr-6108 and Lbr-35 both reached a pre-stationary phase of growth around the 21 h timepoint (T21) (Fig. 2A, B), ATCC 14869 grew about five times slower than Lbr-6108 and Lbr-35 (comparing ODs) in the first 24 h, after which ATCC 14869 started to grow exponentially (Fig. 2C). The growth curves for all *L. brevis* strains in MRS and MRS + MSG is shown in Fig. 2D. The pH of the culture did not decrease below pH 5, which is within the optimum pH range for GAD in LAB [5], at any timepoint.

Detectable amounts of GABA (> 50 µg/mL) were produced by Lbr-6108 as early as T6 with 66 µg/mL and reached a maximum of 520 µg/mL near the pre-stationary phase of growth at T15 in MRS (Fig. 2A, Additional file 1: Table S1). The GABA production by Lbr-6108 correlated with rapid consumption of innate glutamate in MRS. The innate glutamate content was found to be 650–700 µg/mL (0.065–0.07%), which was rapidly utilized by Lbr-6108 to 100 µg/mL after T15, thus showing an 86.0% conversion of glutamate to produce GABA (Fig. 2A). Further, the production of GABA by Lbr-6108 increased upon addition of 10,000 μg/mL (1.0%) free glutamate substrate in the form of MSG in the media (Fig. 2A). A 2.5-fold increase in GABA production was observed at T15 compared to its growth without added glutamate, followed by further utilization of the excess glutamate in the media leading to a conversion of 97.0% of the added glutamate and production of more than tenfold of GABA at 48 h (Fig. 2A). The maximum activity of the GAD enzymatic conversion of glutamate by the strain was seen during the post-stationary phase (T24) when GABA production increased by 39.2% compared to T21 via an increase in glutamate utilization of 34.7% (Fig. 2A). The final concentration of GABA at T48 in MRS + MSG was 6,000 μg/mL or 6 g/L, similar to previous measurements of Lbr-6108 [29].

Noting the GABA producing capabilities of Lbr-6108 from glutamate, the strain was tested for its increased GABA producing capabilities by adding more of the substrate (MSG) to the media. The increase in glutamate in the media from 10,000 μg/mL, to 30,000 μg/mL and 90,000 μg/mL increased the amount of GABA produced by the strain. The amount of GABA produced between T24 and T48 almost doubled with 1.0% MSG, increased 243.0% with 3.0% MSG, and increased 607.0% with 9.0% MSG (Additional file 1: Table S2, Fig. 2E). After T48, the percent of glutamate utilization by Lbr-6108 seemed slowed, although the GABA production was still increasing by T72 in each series. The yield of GABA from available glutamate, calculated by GABA production divided by glutamate utilization, was generally higher with the 10,000 μg/mL MSG formulation, and continued to increase in the longer timepoints (Additional file 1: Table S2, Fig. 2E), with all the bacterial culture media at T24 being at pH 5 (blank media pH 5.5–6). At T48, the bacterial culture in 10,000 μg/mL MSG medium was still at pH 5, but that of 30,000 μg/mL and 90,000 μg/mL MSG-containing media increased to pH 7 and pH 8, respectively.

Strain specific PCR and 16S sequencing was performed on cell pellets directly harvested at T0, T24, and T48 to make sure the harvested strains were pure. No contamination was observed between Lbr-6108 and Lbr-35 (Additional file 1: Fig. S1).

### The time of GABA production is not inherent to GAD operon presence.

Lbr-6108 was the only strain to produce a detectable quantity of GABA as early as 6 h of growth, in both MRS and MRS + MSG (Fig. 2A). Amongst Lbr-35 and ATCC 14869, Lbr-35 had the most similar growth curve to Lbr-6108, whereas the ATCC 14869 grew very slowly in the same media (Fig. 2D). However, detectable quantities of GABA (80–82 µg/mL) produced by Lbr-35 in MRS were observed only at T21–T24, which doubled by T48 (Fig. 2B). In MRS + MSG, GABA production by Lbr-35 did not drastically increase (Fig. 2B). Further, the low level of GABA production by Lbr-35 during the stationary to post-stationary phase of growth in both MRS and MRS + MSG is reflected by limited utilization of glutamate (maximum utilization up to 20.0%) (Fig. 2B). For ATCC 14869, the growth pattern in either MRS or MRS + MSG was very different compared to Lbr-6108 and Lbr-35, and GABA production by ATCC 14869 was comparable to that of Lbr-35, with detectable quantities of GABA only observed between T24–T48 (Fig. 2C). Lbr-6108 and Lbr-35 were sampled for RNA-sequencing because of their similar growth characteristics but differing GABA production capabilities. ATCC 14869 was not sampled for RNA-sequencing, as its slower growth pattern was expected to skew the GAD operon expression results.

### Lbr-6108 expresses genes for GABA production early in fermentation.

**Fig. 3:**
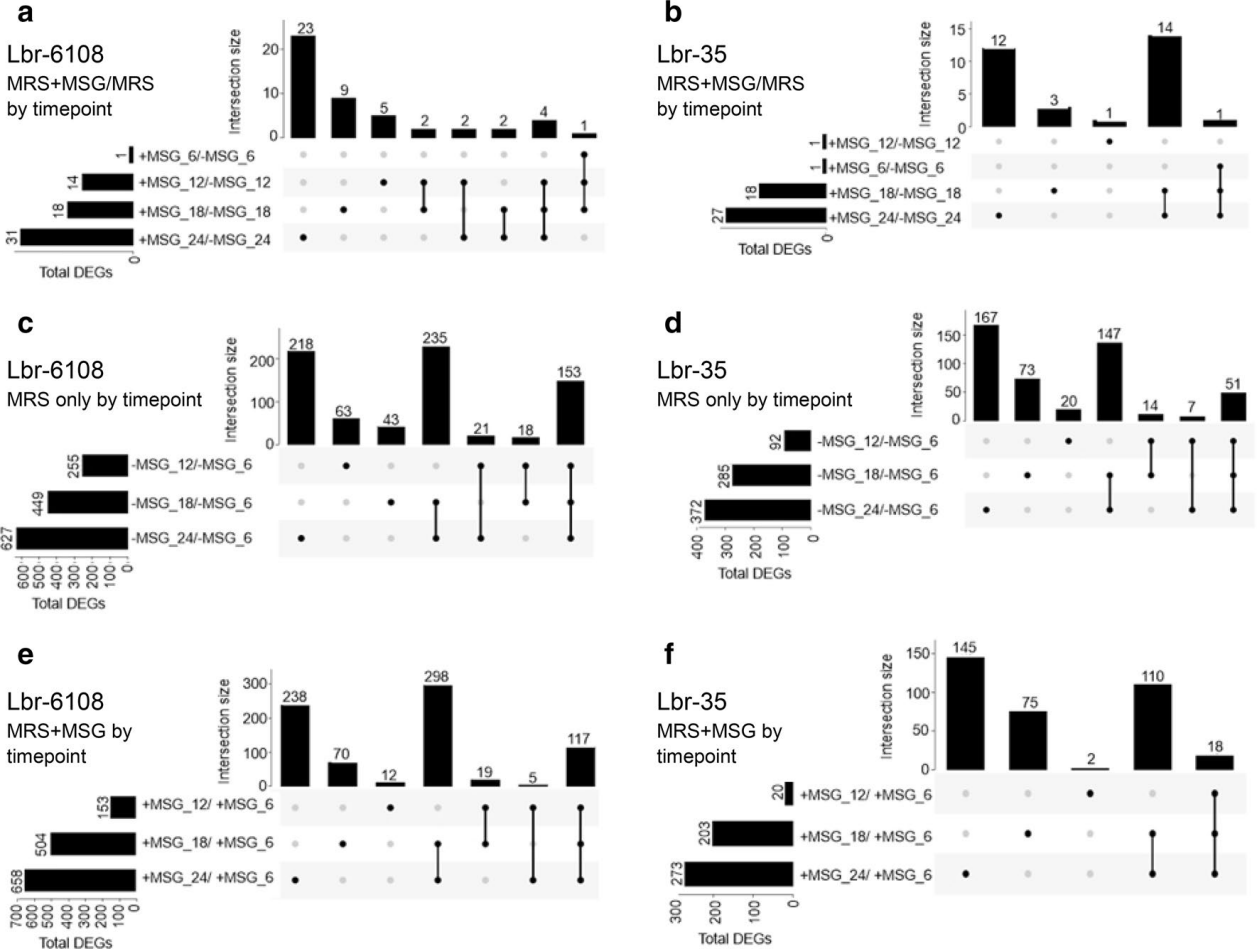
Upset diagram to show significantly differentially expressed genes in *Levilactobacillus brevis* Lbr-6108 and Lbr-35 throughout the experiments. Individual lines are denoted by having MRS only (−MSG) or MRS + MSG (+ MSG) followed by the timepoint number. The top graphs compare the number of significantly expressed genes at the same timepoints but with different media (MRS and MRS + MSG) for (**A**) Lbr-6108 and (**B**) Lbr-35. The middle graphs compare the T6 (pre-log phase) to T12 (mid-log), T18 (pre-stationary) and T24 (stationary) in only MRS for (**C**) Lbr-6108 and (**D**) Lbr-35. The bottom graph compares the T6 (pre-log phase) to T12 (mid-log), T18 (pre-stationary) and T24 (stationary) in MRS + MSG for (**E**) Lbr-6108 and (**F**) Lbr-35

Broad trends in gene expressions were visualized in a principal component analysis plot (Additional file 1: Fig. S2), with replicates of samples mostly clustering together in the same group. However, for Lbr-35 in MRS + MSG, one of the replicates each from T6 and T12 clustered together instead of clustering with their respective replicates. In Lbr-6108, T18 showed the largest differences in gene expression (Table 1). The T18 MRS + MSG samples showed more similarity to gene expressions at T12, while the MRS samples were more similar to T24 (Additional file 1: Fig S2). Figure 3 shows the significantly differentially expressed genes (DEGs) of Lbr-6108 and Lbr-35 individually at different stages of growth and compared to their respective pre-log phase gene expressions at T6. In general, the number of DEGs increased over time, with maximum number of DEGs observed in both strains at T24 compared to T6 (Fig. 3A, B). Lbr-6108 showed more DEGS than Lbr-35 in the earlier growth phase (T12 compared to T6) in MRS and MRS + MSG (Fig. 3C–F). Furthermore, at mid-log phase of growth for Lbr-6108 (T12), 14 genes were differentially expressed in MRS + MSG, whereas at the same growth phase for Lbr-35 (T12) only 2 DEGs were induced in MRS + MSG (Fig. 3E). The fourteen genes of Lbr-6108 comprised of hypothetical proteins, transporters, and other enzymes for sugar fermentation, as well as *thiD2* in the pyridoxal-5′-phosphate (PLP) operon, which highlights subtle expression differences in general growth characteristics between the strains, despite having similar growth curves (Fig. 2A, B).

**Table 1:** Top twenty highest expressed genes in *Levilactobacillus brevis* Lbr-6108 (top) and Lbr-35 (bottom) comparing T18 to T6 in MRS with 10,000 μg/mL monosodium glutamate

Genes	Lbr-6108			Lbr-35		
	log2 fold change	Adjusted p-value	Reads base mean	log2 fold change	Adjusted p-value	Reads base mean
Uronate isomerase (EC 5.3.1.12)	4.90	4.71E−139	10,468	2.29	4.39E−29	1311
NrdR-regulated deoxyribonucleotide transporter, PnuC-like	4.67	5.67E−148	2208	3.22	6.92E−51	1278
Ribonucleotide reductase of class II (coenzyme B12-dependent) (EC 1.17.4.1)	4.41	1.19E−258	79,074	2.86	1.08E−108	52,006
Glucuronide transporter UidB	4.31	5.52E−112	5426	1.56	1.54E−19	477
beta-glucuronidase (EC 3.2.1.31)	4.30	2.64E−165	2277	1.69	2.55E−16	491
Alpha-glucosidase (EC 3.2.1.20)	4.10	4.32E−152	7925	1.72	1.95E−25	1533
Galactokinase (EC 2.7.1.6)	4.01	1.11E−47	1609	1.05	5.53E−06	299
Lactose and galactose permease, GPH translocator family	3.99	2.62E−55	1442	0.39	5.80E−02	362
hypothetical protein	3.97	6.30E−32	152	0.95	1.90E−03	65
Tyrosyl-tRNA synthetase (EC 6.1.1.1)	3.86	1.31E−243	10,243	0.58	9.50E−07	2184
Carbamate kinase (EC 2.7.2.2)	3.86	7.94E−201	7896	1.70	1.30E−23	1,713
Probable glutamate/gamma-aminobutyrate antiporter	3.79	2.11E−164	20,971	− 0.12	5.07E−01	2237
Glutamate decarboxylase GadA (EC 4.1.1.15)	3.74	7.24E−173	26,711	0.17	2.39E−01	2969
Phosphopentomutase (EC 5.4.2.7)	3.64	2.41E−66	6719	− 0.72	4.59E−04	2996
Ornithine carbamoyltransferase (EC 2.1.3.3)	3.64	2.41E−106	26,481	0.83	4.99E−08	9679
Arginine/ornithine antiporter ArcD	3.64	2.05E−113	5413	0.77	3.52E−10	1437
Glutamyl-tRNA synthetase (EC 6.1.1.17) @ Glutamyl-tRNA(Gln) synthetase (EC 6.1.1.24)	3.59	3.90E−152	39,673	0.04	8.67E−01	4886
Phage lysin, glycosyl hydrolase, family 25	3.57	2.01E−17	1011	5.13	2.13E−18	1903
hypothetical protein	3.49	1.36E−161	1036	–	–	–
Purine nucleoside phosphorylase (EC 2.4.2.1)	3.48	9.00E−58	3140	− 0.43	5.93E−02	2133
Aggregation promoting factor	2.80	7.03E−15	37,746	5.84	1.04E−25	22,462
Phage lysin, glycosyl hydrolase, family 25	3.57	2.01E−17	1011	5.13	2.13E−18	1903
lipoprotein precursor (putative)	3.16	7.83E−54	990	4.08	3.75E−79	1987
NLP/P60 family protein	1.40	7.51E−14	1243	3.85	4.25E−45	5309
NrdR-regulated deoxyribonucleotide transporter, PnuC-like	4.67	5.67E−148	2208	3.22	6.92E−51	1278
Phage lysin, glycosyl hydrolase, family 25	2.90	3.22E−122	22,673	2.88	3.06E−47	12,481
Beta-lactamase class C-like and penicillin binding proteins (PBPs) superfamily	2.20	2.22E−44	7114	2.87	2.95E−47	8280
Ribonucleotide reductase of class II (coenzyme B12-dependent) (EC 1.17.4.1)	4.41	1.19E−258	79,074	2.86	1.08E−108	52,006
peptidoglycan lytic protein P45	2.32	5.18E−79	4020	2.85	2.14E−86	3066
Ribonucleotide reductase of class Ib (aerobic), beta subunit (EC 1.17.4.1)	3.10	1.75E−67	57,256	2.80	2.80E−138	50,915
Streptococcal hemagglutinin protein	2.60	8.15E−24	2848	2.72	5.81E−22	3186
Bifunctional autolysin Atl/*N*-acetylmuramoyl-l-alanine amidase (EC 3.5.1.28)/ endo-beta-*N*-acetylglucosaminidase (EC 3.2.1.96)	1.64	1.61E−22	11,980	2.69	1.48E−38	10,461
Ribonucleotide reductase of class Ib (aerobic), alpha subunit (EC 1.17.4.1)	2.52	2.47E−108	55,842	2.64	5.47E−118	46,441
Aggregation promoting factor	0.50	5.01E−03	684	2.60	4.12E−20	698
Aggregation promoting factor	0.72	3.32E−03	1607	2.59	2.95E−24	890
tRNA-5-carboxymethylaminomethyl-2-thiouridine(34) synthesis protein MnmE	2.45	5.57E−70	3144	2.56	8.51E−48	3573
DNA topoisomerase IV subunit A (EC 5.99.1.3)	1.39	8.11E−56	5955	2.49	6.22E−90	13,461
Succinate-semialdehyde dehydrogenase [NAD] (EC 1.2.1.24); Succinate-semialdehyde dehydrogenase [NADP +] (EC 1.2.1.79)	2.73	5.84E−131	4798	2.37	2.51E−66	4039
tRNA-5-carboxymethylaminomethyl-2-thiouridine(34) synthesis protein MnmG	2.63	1.75E−52	4279	2.36	3.10E−31	5697
Uronate isomerase (EC 5.3.1.12)	4.90	4.71E−139	10,468	2.29	4.39E−29	1311

**Fig. 4:**
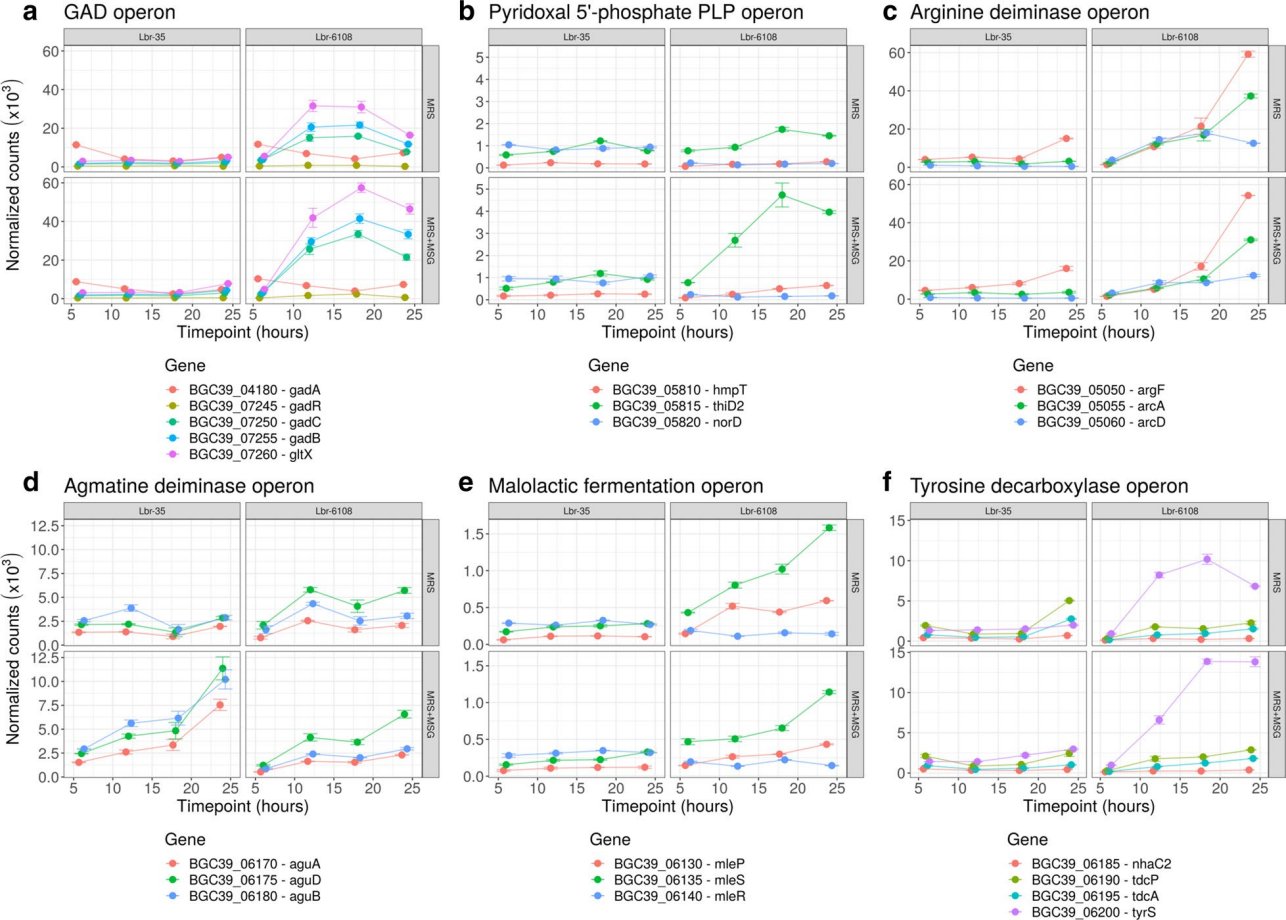
Gene expression of acid resistance operons in *Levilactobacillus brevis* Lbr-6108 and Lbr-35. Each quadrant shows normalized RNA expression for genes during growth for Lbr-6108 and Lbr-35 in MRS and MRS with 10,000 μg/mL MSG. Gene symbols are defined in table S2 and as follows: **A** *gadA*, Glutamate decarboxylase (EC 4.1.1.15); *gadR*, Transcriptional regulator; *gadC*, Glutamate/gamma-aminobutyrate antiporter; *gadB*, Glutamate decarboxylase (EC 4.1.1.15); *gltX*, Glutamyl-tRNA synthetase (EC 6.1.1.17; EC 6.1.1.24). **B** *hmpT*, Substrate-specific component HmpT of predicted hydroxymethylpyrimidine ECF trans-porter; *thiD2*, Novel pyridoxal kinase, thiD family (EC 2.7.1.35); *norD*, Transcriptional regulator of pyridoxine metabolism / Pyri-doxamine phosphate aminotransferase (EC 2.6.1.54). **C** *argF*, Ornithine carbamoyltransferase (EC 2.1.3.3); *arcA*, Arginine deimi-nase (EC 3.5.3.6); *arcD*, Arginine/ornithine antiporter. **D** *aguA*, Agmatine deiminase (EC 3.5.3.12); *aguD*, Agmatine/putrescine antiporter, associated with agmatine catabolism; *aguB*, Putrescine carbamoyltransferase (EC 2.1.3.6). **E** *mleP*, Malate permease; *mleS*, Malolactic enzyme (EC 4.1.1.101); *mleR*, Malolactic regulator. **F** *nhaC2*, predicted tyrosine transporter, NhaC family; *tdcP*, Predicted tyrosine transporter, GadC family; *tdcA*, l-tyrosine decarboxylase (EC 4.1.1.25); *tyrS*, Tyrosyl-tRNA synthetase (EC 6.1.1.1)

**Fig. 5:**
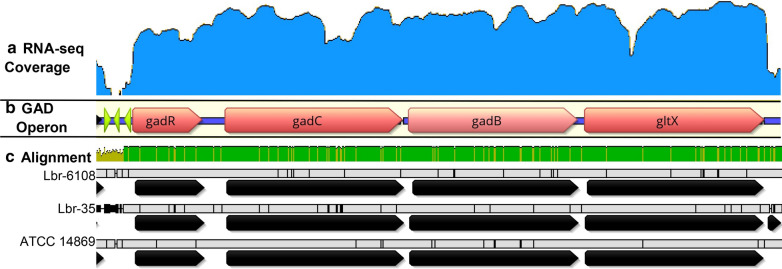
Summary of RNA expression in the *Levilactobacillus brevis* GAD operon. **A** The RNA sequencing read coverage is indicated by the blue graph in log form for *L. brevis* Lbr-6108 in MRS with 10,000 μg/mL MSG at T18. **B** The *L. brevis* GAD operon is denoted by the orange arrows and putative promoters denoted by lime green arrows. **C** The DNA sequence pairwise alignment of *L. brevis* Lbr-6108, Lbr-35, and ATCC 14869 is shown with green showing 100% identity and yellow representing 30–99% identity. Individual GAD operon sequence polymorphism is shown by black lines in the grey bars according to strain designation on the left

The GAD operon regulation vastly differed between Lbr-6108 and Lbr-35 in both media, despite the cultures being sampled at similar points in the growth curve (Fig. 4A, Additional file 1: Table S3). Comparing T12 to T6 in Lbr-6108, the fold change of gene expression for *gadB* significantly increased 2.47 × and 3.28 × in MRS and MRS + MSG, respectively (Fig. 4A). The *gadA* expression did not change significantly between any timepoint, indicating that *gadB* is the major glutamate decarboxylase in Lbr-6108 (Fig. 4A). The *gadB* expression continued to increase at T18 but decreased at T24 (Fig. 4A). As expected, *gadC* gene expression increased throughout the experiment similarly as *gadB*, and its expression in MRS + MSG were nearly double that of MRS (Fig. 4A). The *gadR* was upregulated from T12–T18 almost two-fold in MRS but was upregulated more than four-fold in MRS + MSG (Fig. 4A). Importantly, the *thiD* family pyridoxal kinase gene *thiD2,* which catalyzes the phosphorylation of pyridoxal to PLP, increased in expression similarly to the GAD operon, confirming the ability of Lbr-6108 to produce the cofactor PLP which is required for GAD catalytic function [33] (Fig. 4B). Interestingly, the RNA expression mapping indicated that *gadR*, *gadC*, *gadB*, and *gltX* are all co-transcribed as the read coverage is similar and is maintained across all the genes (Fig. 5A). The GAD operon showed expected expression patterns from the RNA sequencing data, as cDNA read coverage for the GAD operon started at the predicted promoter region directly upstream of *gadR* (Fig. 5B).

Some activity of the GAD operon was expected in Lbr-35 since the GAD operon is highly identical to Lbr-6108 (Fig. 5C), but surprisingly there were no significant changes in expression from T6-T24, except for the downregulation of *gadA* at T18-MRS, T12-MRS, and T18-MRS + MSG (Fig. 4A). The GAD operon expression pattern in Lbr-35 did not differ when grown in the absence or presence of additional MSG in the MRS media. Amongst the DEGs of Lbr-35 grown in MRS + MSG and MRS only, 18 (Fig. 3C) and 51 (Fig. 3B) genes respectively, were differentially expressed at all timepoints throughout the growth of the strain past mid-log phase (T12, T18, and T24). Of those DEGs, genes in the agmatine deiminase pathway, including agmatine deiminase *aguA* (enzyme class (EC) 3.5.3.12) and agmatine/putrescine antiporter *aguD*, were upregulated for both Lbr-6108 and Lbr-35 (Fig. 4C). In Lbr-6108, the arginine deiminase operon was highly expressed only at T24 of growth in both MRS and MRS + MSG (Fig. 4D). The F_O_F_1_-ATPase operon, which consists of multiple subunits of ATP synthase chains, did not significantly change in either strain except for the ATP synthase epsilon chain (EC 3.6.3.14) in Lbr-6108 at several timepoints of MRS and MRS + MSG (data not shown). At T18 in MRS + MSG, the timepoint of highest GAD operon expression in Lbr-6108, Lbr-35 is instead significantly expressing multiple phage lysin related genes, genes for transporter proteins, and three aggregation promoting factors (Table 1).

### Other metabolic pathways for acid resistance and energy generation are expressed concurrently with the GAD operon in Lbr-6108.

In Lbr-6108, 153 and 117 DEGs were observed between the mid-log phase at T12 until the stationary phase at T24 in MRS and MRS + MSG, respectively (Fig. 3). Part of the 153 DEGs found in MRS were genes of the highly expressing arginine deiminase operon, which includes Arginine/ornithine antiporter *arcD*, Ornithine carbamoyltransferase *argF* (EC 2.1.3.3), and Arginine deiminase *arcA* (EC 3.5.3.6) (Fig. 4D). Malolactic fermentation is known to reduce acidity [34], and indeed the malolactic enzyme *mleS* gene is increased in Lbr-6108 throughout the different timepoints (Fig. 4E). A malate permease *mleP* of the malate fermentation operon was slightly upregulated at T12 in both MRS and MRS + MSG (Fig. 4E). Similarly, the 117 DEGs for Lbr-6108 in MRS + MSG (Fig. 3E) included upregulation of genes in the agmatine deiminase, malolactic fermentation, and the tyrosine decarboxylase operons (Fig. 4C–F). Importantly, in the tyrosine decarboxylase operon only the tyrosyl-tRNA synthetase gene *tyrS* showed high induction rate while tyrosine decarboxylase gene *tdcA* was not induced at any timepoints in any of the strains or conditions tested. Further, the arginine deiminase enzyme were always upregulated and to the same extent as when grown in both media (Fig. 4D). Importantly, Many of these genes upregulated in both MRS and MRS + MSG are associated with different acid resistance mechanisms in Lbr-6108 [35]. In general, the l- and d-lactate dehydrogenase genes were not significantly up- or downregulated in Lbr-6108, indicating that there was a steady production of lactic acid in the cultures (data not shown).

**Fig. 6:**
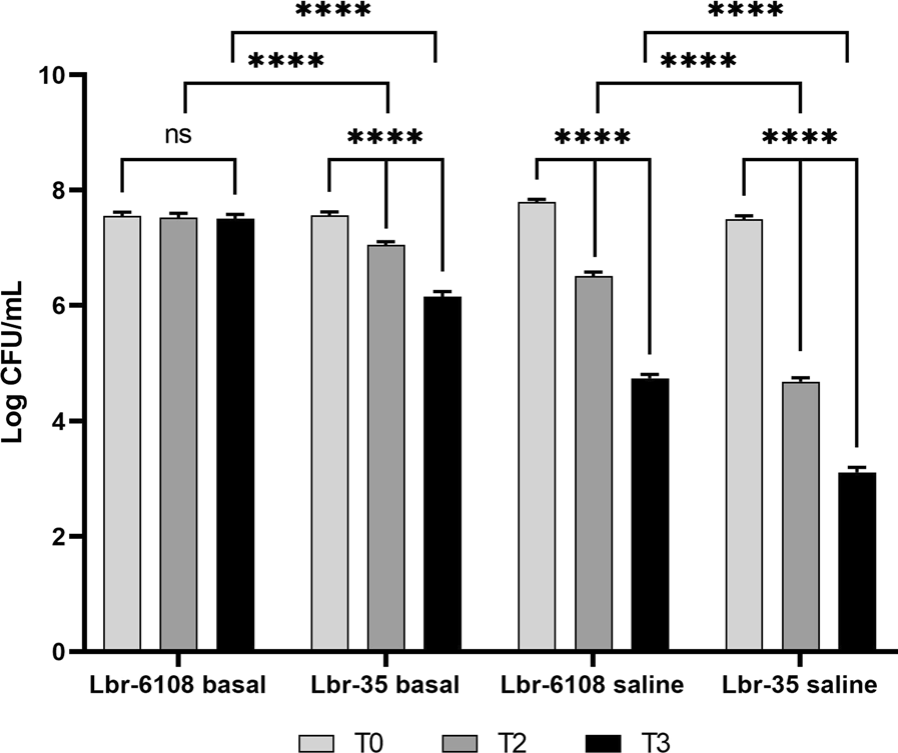
Acid resistance of stationary-phase cultures of *Levilactobacillus brevis* Lbr-6108 and Lbr-35. The survival of the strains was determined by overnight culture in MRS followed by acid challenge at pH 3.0 in a basal medium solution and pH 3.0 in 0.5% saline solution. All cultures were grown to stationary phase overnight at 37 °C. Viable cell counts were assessed immediately, and recovery rates were determined at 2 h and 3 h following exposure to acidic environment. Values represent the mean of four biological replicates plated in quadruplicate, and error bars represent the standard error. Bars with **** differ significantly (p < 0.0001) according to two-way ANOVA with Tukey’s multiple comparisons test

Since many of these metabolic groups are involved in acid resistance, Lbr-6108 was tested for an increased acid tolerance compared to Lbr-35. Both strains were exposed to acidified media for various lengths of time before enumeration of viable cells. The viable cell counts for Lbr-6108 was reduced by a statistically insignificant amount (< 1.0%) of its initial concentration after 3 h of exposure to basal media with pH 3.0. In contrast, Lbr-35 was reduced significantly by two logs in the same exposure parameters (Fig. 6). A saline-based medium without carbohydrates was used to assess if the acid resistance was due to increased uptake of carbohydrates, amino acids, peptides, or other nutrients in the basal medium. Indeed, both strains were significantly reduced when exposed to pH stress in the saline solution (Fig. 6), although Lbr-6108 was significantly higher than Lbr-35. This indicates that Lbr-6108 induces acid resistance by metabolizing carbohydrates.

## Discussion

Although GABA is relatively abundant in plant based foods such as brown rice germs and sprouts, spinach, barley and bean sprouts (300–720 nmol/g dry weight), foods containing GABA cannot meet consumers’ needs based on certain placebo-controlled studies [36, 37]. A systematic review of past clinical trials using natural or biosynthetic GABA showed that effective doses for stress reduction are not a standard concentration, but rather a range from 2.01 to 100 mg, with lower doses affecting autonomic markers and higher doses affecting central markers, and that an extended intake instead of a single dose is necessary for efficacy [38]. This highlights the importance of safe sources of GABA that can be regularly consumed. Microorganisms, including LAB, are an important source of GABA [1, 39–42], and the GABA producing capability amongst LAB like lactobacilli varies between species and strains [43–47]. More recently, high GABA producing lactobacilli strains have been isolated to meet consumer needs [45, 48]. In this manuscript, we sought to further investigate strain-specific mechanisms for GABA production in *Levilactobacillus brevis*.

The growth experiments showed Lbr-6108 started producing GABA earlier during fermentation than the other two strains tested, within 6 h of fermentation start. Many publications show that the optimum time for GABA output is after several days of growth, typically at least 48 h [5, 29, 49]. Indeed, Lbr-35 and ATCC 14869 produced considerable amounts of GABA only at 24–48 h. The increase of GABA was consistent with the decrease in glutamate concentration in the experiments. The intrinsic glutamate level of MRS, the synthetic medium used in the growth experiment, is similar to the mean daily intake of glutamate, which is around 15 g for meat-eaters, fish-eaters, vegetarians, and vegans [50]. MSG, the sodium salt of glutamate, is used widely in commercial food industry as a flavor enhancer with about an average daily consumption of 0.6 g (range 0.3–2.0 g/day) in the USA and Europe, and 2–3 times that in East and Southeast Asian countries [52–54]. Glutamate acts as an excitatory neurotransmitter, however excess glutamate may lead to increased excitation responses within the cells to which it binds, leading to cell death in a process known as “excitotoxicity” [55]. In fact, the glutamate concentration in a final food should not exceed 10 g/Kg in the European Union [51]. Thus, whereas MSG as a flavor enhancer is beneficial for the food industry, the ubiquitous use of this food-additive could have deleterious consequences to public health [54, 56, 57]. Since the mean gastrointestinal transit time for the average healthy adult human is around 30 h [52], rapid activation of the GAD operon by Lbr-6108 may allow for beneficial conversion of the dietary sodium salt of glutamate to GABA in vivo.

The growth and RNA-sequencing experiments confirmed that Lbr-6108 produced more GABA than other strains with similar growth patterns and syntenous GAD operons, and that the GAD operon was upregulated accordingly. Lbr-35 was used as a negative control to assess various transcriptomic strategies recently reviewed that could explain the differences of GABA production [1]. Simultaneous expression of both GAD genes in *L. brevis* will increase GABA production, but only the *gadB* gene showed differential expression in Lbr-6108. A recent study showed that the increase of GABA from an isolate of *L. brevis* from Chinese light-aroma type liquor could be attributed in part to a hyperactive *gadR* gene [5]. Likewise, increasing the expression *gadC* allows for more substrate to be brought in from the extracellular environment. Further, upregulation of the PLP operon ensures enough cofactor is available for the decarboxylation process. The genes in the GAD operon did not seem constitutively expressed in Lbr-6108, as their expression fluctuated with glutamate concentration and over time. Of note, however, the RNA-sequencing results showed the GAD operon and *gltX* is co-transcribed, which confirms and expands upon findings determined from co-expression analysis by qPCR of *gadC* and *gadB* from previous research [4]. Repressing key genes for pathways that would redirect glutamate from the GAD operon, such as *gltX* for translation or *gabT* to shunt glutamate to the tricarboxylic acid cycle or decreasing the F_O_F_1_-ATPase system, would also increase GABA output. Each of these hypotheses were evaluated as to why Lbr-6108 has increased GABA producing capabilities compared to Lbr-35, but none were identified as the direct genetic or transcriptomic reason why Lbr-35 differed so much from Lbr-6108 was identified. The GAD operon expression in Lbr-35 did not increase during fermentation even with MSG, and *gadA* did not change expression in any condition for Lbr-6108 or Lbr-35. Neither strain has a *gabT* homolog, and *gltX* only increased in Lbr-6108 despite overall sequence homology. The F_O_F_1_-ATPase system did not significantly increase in any condition in either strain. Interestingly, the *thiD2* gene in the PLP operon had significantly increased expression in Lbr-6108 with the addition of MSG, despite being similar in both strains with just MRS. Future studies directly quantifying PLP in *L. brevis* would be useful in further analyzing its transcriptional effects. A putative promoter region identified upstream of the GAD operon in Lbr-6108 could increase GAD operon expression, but further analysis is needed. Altogether, this highlights the complexity of global regulation for GABA in lactobacilli beyond GAD operon activation.

The RNA-sequencing also revealed that Lbr-6108 activated several other acid resistance pathways concurrently with the GAD operon. Although the pH was qualitatively measured in the experiments to ensure the strains remained in the optimum GABA-producing pH for *L. brevis*, further experimentation titrating high and low pH values, especially in consideration of the pH levels of the large and small intestines, may reveal great transcriptional effects. Nonetheless, the rapid production of GABA was accompanied by activation of several other acid resistance mechanisms, including arginine deimination and malate fermentation. It is important to highlight that the actual tyrosine decarboxylase gene, which would catalyze the biogenic amine tyrosine and is toxic for hosts, was not upregulated, but rather the tyrosyl-tRNA synthase. These processes are not only important for acid resistance, but also for electrogenic exchange to create proton motive force for energy generation [35]. It is tempting to hypothesize that Lbr-6108, which was isolated from infant fecal microbiota, representing the complex infant gut microbiota, is accustomed to energy and substrate scavenging, so it activates a variety of strategies early in the growth phase or during the stationary phase, in the case of the arginine operon. This may in help increase commensal organisms in the immature infant microbiota, as GABA was recently shown to be an essential growth factor for non-culturable human microbes [53]. In contrast, Lbr-35 may be accustomed to operating under resource-limited conditions of food environments and would only produce excess GABA under severe acid stress or when starved for energy. Further experimentation is required to assess this hypothesis of GABA production as a by-product of a diverse set of resource acquisition strategies.

Lbr-35 differed considerably from Lbr-6108 in the response to glutamate in the growth media, despite having similar growth patterns. Recent research has also shown that GABA production in *L. brevis* is not contingent on optimized growth [54], however screening of various carbohydrates may increase GABA production in Lbr-35. We found that no genes in the GAD operon were significantly expressed in Lbr-35 in MRS with or without MSG. The only metabolic pathways for acid resistance that was upregulated in the Lbr-35 RNA-sequencing was for agmatine metabolism, which increased with the addition of 10,000 μg/mL MSG. Agmatine, like GABA, is an alternative nitrogen source used by bacteria [55], and a precursor of putrescine, which is the most abundant biogenic amine found in wine [56]. Additionally, Lbr-35 expressed genes related to stress response, including aggregation promoting factors (APFs), which have been shown to be a desirable characteristic of probiotic lactobacilli to aid in competitive exclusion of pathogens [57, 58]. Further, upregulation of APFs is important for biofilm formation and contributes to gastric/small intestinal juice survival and adhesion to epithelial cells [57]. This implies that Lbr-35 activates an alternative response to fermentation stress that focuses on aggregation and biofilm formation, rather than nutrient metabolism and de-acidification in Lbr-6108. While strain-specific strategies for GABA production is the subject of many studies, functional analysis of low-GABA producing strains also warrants further research.

The rapid and increased production of GABA by microbes like Lbr-6108 could have health benefits for the host microbiota-gut-brain axis. The role of microorganisms in the gut for normal brain development, function, physiology, and behavior has been identified, with some of the most convincing evidence emerging from germ-free rodent models and various intervention studies including diet, prebiotics, and probiotics [17]. Indeed, GABA-producing *Bifidobacterium dentium* ATCC 27678 reduced visceral hypersensitivity in rats [59]. As mentioned in the introduction, a seminal study showed GABA-producing *Lacticaseibacillus rhamnosus* JB-1 decreased stress-induced biomarkers in the brain through regulation of the GABAergic system [25]. Interestingly, the effect on anxiety-related behavior stopped after a vagotomy was performed, which indicates that the microbially-produced GABA had a direct effect on the brain mediated through the vagus nerve. In addition to effects on stress-related behavior and brain function, microbial GABA production has also been associated with improved memory and sleep quality [60, 61]. Communication along the microbiota-gut-brain axis is not only mediated by microbial-GABA production, as many studies using typical probiotic species showed benefits related to the gut-brain axis without focusing on GABA. For example, *Lacticaseibacillus paracasei* Lpc-37 was shown to improve different stress metrics in a mouse trial [62] and human clinical trial [63], while regular consumption of milk with GABA from *Lacticaseibacillus casei* strain Shirota and *Lactococcus lactis* YIT 2027 reduced the risk of hypertension in elderly [64, 65]. Screening novel probiotics for optimal effector functions could increase the likelihood and impact of health benefits. Indeed, Lbr-6108 was screened against other intestinal bacterial isolates for high GABA output and has shown a variety of health benefits in vitro and in rodents [29–31]. In applications where genome modification is not yet widely adopted, organisms that naturally produce high amounts of effector molecules like GABA, and are recognized as safe, are primary candidates as supplemental solutions.

## Conclusion

In the present study, RNA sequencing under physiologically relevant laboratory conditions was used to assess the global regulatory aspects of GABA production. This revealed that transcription of the *Levilactobacillus brevis* GAD operon is not contingent merely on the presence on *gadR*, as strains that have similar GAD operon sequences do not produce GABA under the same conditions. As such, high GABA production, especially early in the growth phase, was confirmed to be strain-specific. Having established the potent GABA output from Lbr-6108, further studies are warranted to investigate the microbial influence on brain function and behavior. In addition, more robust clinical trials are required to determine if supplementation with GABA-producing bacterial strains like Lbr-6108 can influence behaviors mediated by GABAergic signaling such as stress and anxiety. Development of strains like Lbr-6108 as psychobiotics to modulate the neural excitatory-inhibitory balance, mood, cognitive functions, learning and memory processes could be promising as complementary remedies for the management of mental health.

## Materials and methods

### Bacterial strains and media

Frozen glycerol stock vials of *Levilactobacillus brevis* Lbr-6108 (ATCC safe deposit (SD) SD-7285) originally isolated from human intestines, *Levilactobacillus brevis* ATCC 14869 originally isolated from feces, and commercial probiotic *Levilactobacillus brevis* Lbr-35 (ATCC SD-5214) were obtained from the Danisco Global Culture Collection. The strains were grown in De Man-Rogosa-Sharpe (MRS) broth (Becton Dickinson (BD), Franklin Lakes, NJ, USA) at 37 °C ± 1 °C under anaerobic conditions (BBL Gas Pak and BD GasPak EZ container systems, Becton Dickinson, Cockeysville, MA, USA).

### Bacterial culturing and harvesting

Overnight cultures (16–18 h) of *L. brevis* Lbr-6108, Lbr-35, and ATCC 14869 were inoculated in 15 mL disposable culture tubes (p/n 14-961-27; Fisher, Hanover Park, IL, USA) containing 3 mL of media to reach an optical density (OD) of ~ 0.100 measured at 600 nm using a spectrophotometer (Genesys 20; Thermo Fisher, Waltham, MA, USA). For each strain, two types of media were used; MRS broth (BD) without any other supplementation (further denoted as MRS) and MRS broth supplemented with 10,000 μg/mL (equivalent to 10 g/L) of l(+)-glutamic acid monosodium salt monohydrate (p/n 119940010; Acros Organics, Morris Plains, NJ, USA) (further denoted as MRS + MSG). The OD measurement for timepoint zero (T0) was measured shortly after inoculation of the overnight culture into disposable culture tubes containing 3 mL media. Cultures for all except ATCC 14869 were harvested at the initial timepoint (T0) and then every 3 h post-inoculation until 24 h, and then at 48 h post-inoculation and the OD was measured just before harvesting. For ATCC 14869, the cultures were harvested after measuring the OD at different time points of 9 h, 24 h and then at 48 h post-inoculation because of its slower growth pattern. For each harvest timepoint separate sets of culture tubes were prepared, so that only the set of tubes for the designated time points were removed from the incubator for harvesting, without disturbing the culture growth. All culture tubes with strains growing in MRS or MRS + MSG were placed in the 37 °C ± 1 °C incubator under anaerobic conditions using anaerobic GasPak EZ container systems (p/n 260001, BD). Samples were removed from the incubator at respective timepoints and the ODs were measured at 600 nm. All OD measurements were recorded, and a growth curve was plotted using GraphPad Prism v. 9.0.1 (La Jolla, CA, USA).

Each timepoint sample set included a blank (cell free media) and the inoculated strains in duplicates in both MRS and MRS + MSG. At each timepoint, the cell-free supernatant was harvested for metabolomic analyses. Cultures (3 mL) were centrifuged at 1500×*g* for 5 min, the supernatant was filtered through GD/X 25 mm syringe filter (polyvinylidene difluoride filtration medium, 0.2 µm, GE Healthcare Life Sciences, Cytiva, Marlborough, MA) and 1 mL of cell free supernatant was aliquoted into sterile cryogenic vials and frozen at − 80 °C until analyzed. The pH for each sample set including the blanks was measured at each harvesting timepoints (p/n: 13-640-516, Fisher).

The cell pellets from all harvested timepoint samples of Lbr-6108 and Lbr-35 were treated with RNA protect (p/n: 76526, Qiagen, Hilden, Germany) as per manufacturer’s protocol. Briefly, 3 mL of RNA protect was added to the pellets and resuspended by vortexing. The RNA protect resuspended cell pellets were incubated at room temperature for 5 min, and then centrifuged at 5000×*g* for 10 min. The supernatant was discarded, and the cell pellet was frozen at − 80 °C. The frozen cell pellets of Lbr-6108 and Lbr-35 cultures grown in MRS and MRS + MSG from timepoints 6 h (pre-log phase), 12 h (mid-log phase), 18 h (pre-stationary phase) and 24 h (stationary to post-stationary phase) of growth were analyzed by RNA-sequencing (GENEWIZ NJ Lab, South Plainfield, NJ, USA). Further timepoint references are denoted by T6–T24 after inoculation.

In a separate experiment, similarly as above, Lbr-6108 only was grown in MRS containing 0 μg/mL, 10,000 μg/mL, 30,000 μg/mL, and 90,000 μg/mL MSG to determine the influence of glutamate concentration on GABA production. Cell free culture supernatants were harvested at times 5 h (pre-log phase), 24 h (stationary phase), 48 h and 72 h (post-stationary phase) for quantitation of GABA and glutamate.

To test the acid resistance of Lbr-6108 and Lbr-35, the strains were grown in saline solutions and basal media. Aliquots of ~ 10 μL scraped from the top of frozen glycerol stocks of Lbr-6108 and Lbr-35 were transferred to 15 mL conical centrifuge tubes (p/n 07-200-886; Thermo Fisher Scientific, Waltham, NJ, USA) containing 10 mL MRS broth and grown overnight (18 h) at 37 °C. Overnight cultures were reactivated once by transferring 0.1 mL aliquots of overnight culture to fresh MRS broth and were grown to stationary phase (21–24 h) at 37 °C. Cultures were pelleted by centrifugation at 5000 rpm for 2 min, washed once with Butterfield’s Phosphate Buffer (p/n R23701; Thermo Fisher Scientific) and resuspended in 2 mL Butterfield’s Phosphate Buffer. Acid survival experiments were performed using a saline solution with 5 g/L sodium chloride (p/n S640-3; Thermo Fisher Scientific) and a basal medium solution with 10 g/L peptone (p/n 211681; Thermo Fisher Scientific), 2 g/L yeast extract (p/n 288620; Thermo Fisher Scientific), 5 g/L sodium chloride, 2 g/L diammonium citrate (p/n JT0682-1; VWR, Radnor, PA, USA), 0.2 g/L magnesium sulfate (p/n MK607012; VWR, Radnor, PA, USA), 2 g/L dipotassium hydrogen phosphate (p/n P3786; Sigma-Aldrich, St. Louis, MO, USA) and 20 g/L glucose (p/n G8270; Sigma-Aldrich). Both solutions were adjusted to pH 3.0 with 6 N HCl (p/n BDH7204-2; VWR). In each experiment, 25 mL of the saline and basal medium solutions were inoculated with 25 μL of the resuspended pellet (~ log 10.6 CFU) for both strains. Samples were mixed, and tenfold serial dilutions were immediately prepared for T0 plating in quadruplicate. Samples were held at 37 °C and additional tenfold dilutions were prepared at 2 h and 3 h timepoints. Samples were pour plated with MRS agar and incubated at 37 °C for 72 h. Subsequently, colonies were counted to assess survival under acidic conditions.

### PCR confirmation of species and strain identity

Using the cell pellets from the harvest timepoints of T0 and/or T24 and/or T48, PCRs were performed using primers designed from 16S rRNA gene and sequenced to confirm the identity of the species (Additional file 1: Fig. S1). Strain-specific PCRs were performed with primers designed to differentiate between Lbr-6108, Lbr-35 and ATCC 14869, with Lbr-6108 to yield a 130 base pair (bp) amplification product using the primers Lbr-6108_Hyp_F (5′-GAACTTCATCAGTAGTGCGTTA-3′) and Lbr-6108_Hyp_R (5′-TGTTGGTCTTCGATATAGGTTAG-3′), while Lbr-35 and ATCC 14869 to yield a PCR product of 267 bp with the same primer set. Another Lbr-6108 strain identifying primer set, EP_F (5′-ACGTCTGGTTATAGCTCATCA-3′) and EP_R (5′-TAGTTTATCGACCGAGCCTT-3′) was designed to yield a 213 bp amplification product from Lbr-6108, and a 339 bp product from Lbr-35 and ATCC 14869. The PCRs were performed according to manufacturer’s specification of AmpliTaq Gold 360 polymerase (p/n 4398881, Thermo Fisher) and visualized using 2% ethidium bromide E-gels (p/n G501802, Thermo Fisher).

### Metabolite profiling

#### Chemicals and standards for metabolomics

High performance liquid chromatography (HPLC) grade acetonitrile and methanol were purchased from Thermo Fisher Scientific (Waltham, MA, USA). Formic acid (> 98.0%), GABA (≥ 99.0%), γ-aminobutyric acid-2,2,3,3,4,4-d_6_ (97.0% atom D), l-glutamic acid (> 99.0%) and l-glutamic acid-2,3,3,4,4-d_5_ (97.0% atom D, 98.0%) were obtained from Sigma-Aldrich. The AccQ Tag Ultra derivatization kit were purchased from Waters (Milford, MA, USA). Water was purified in a Milli-Q water purification system from Millipore (Burlington, MA, USA).

#### Liquid chromatography–mass spectrometry (LC–MS) analysis

A stock solution was prepared by weighing out and dissolving GABA (1000 µg/mL) and l-glutamic acid (2000 µg/mL) in water. Standard solutions for GABA and l-glutamic acid were made by serial dilution in the concentration range 10–1000 µg/mL and 20–2000 µg/mL, respectively. A solution of isotopically labeled internal standards (200 μg/mL) was prepared in water. 200 µL of each standard level was spiked with 50 µL internal standard solution and 800 µL of 0.1% formic acid in methanol was added. The standard solutions were subsequently derivatized using 6-aminoquinolyl-*N*-hydroxysuccinimidyl carbamate (AQC), as described below.

To 100 µL thawed cell free supernatant, 900 µL water was added. 200 µL of the diluted sample was spiked with 50 µL internal standard solution and precipitated with 800 µL 0.1% formic acid in methanol. The precipitation of protein was increased by cooling at − 18 °C for 1 h. The samples were centrifuged for 5 min at 4700 rpm at 4 °C. The supernatant was subsequently AQC-derivatized, as described below. Samples were prepared in duplicate. 20 µL of the standard solution or sample supernatant was mixed with 60 µL of AccQ Tag Ultra borate buffer and 20 µL AccQ Tag reagent for AQC derivatization. The reaction was run for 10 min at 55 °C [66].

The LC–MS analysis of GABA and l-glutamic acid was performed on an Agilent HPLC 1200 series system equipped with degasser, binary pump, microwell plate autosampler, thermostat for autosampler, and thermostat column compartment (Agilent Technologies, Santa Clara, CA, USA). The HPLC was coupled on-line with a triple quadrupole mass spectrometer with heated electrospray interface from Thermo Scientific model TSQ Vantage. In ESI positive mode, AQC-GABA and AQC-GABA-d_6_ generated protonated ions, [M + H]^+^ *m*/*z* 274.1 and 280.1, respectively. AQC-Glutamic acid and AQC-Glutamic acid-d_5_ generated protonated ions [M + H]^+^ *m*/*z* 318.1 and 323.1, respectively.

The compounds were separated on an Atlantis^®^ dC18 3 µm 2.1 × 100 mm column (Waters). Mobile phase A was 0.1% formic acid in water and mobile phase B was 0.1% formic acid in acetonitrile. The linear separation gradient was 0–1 min (95.0% A), 5 min (85.0% A), 7 min (70.0% A), 8 min (5.0% A), 8–10 min (5.0% A), 10.1–15 min (95.0% A). The flow was kept at 0.4 mL/min. The autosampler was set at 5 °C and 1 µL of the sample/standard was injected for analysis. The column oven was set at 30 °C.

### Genome and RNA sequencing

#### Genome sequencing

Both Lbr-6108 and ATCC 14869 have publicly available genomes, however, a high-quality draft genome for Lbr-35 was sequenced and assembled as previously described [67]. Briefly, genomic DNA was sequenced using both paired-end 250 nucleotide sequencing on an Illumina MiSeq (Illumina, San Diego, CA, USA) and on a GridION X5 sequencer (Oxford Nanopore Technologies, Oxford, UK) at the Roy J. Carver Biotechnology Center, University of Illinois at Urbana-Champaign. After quality control, base calling and trimming, a hybrid assembly was performed using Unicycler assembler v 0.4.3 [68]. The genome was annotated in PATRIC using RASTk [69].

#### Comparative genomics

Genomic comparison between the strains was performed using PATRIC Bioinformatics Resource Center [70]. The genome alignment, GAD gene pairwise alignments and percent sequence identity between GAD genes of the different strains were calculated using Geneious (Geneious Prime^®^ 2019.2.1, Biomatters, Auckland, New Zealand).

#### RNA sequencing and analysis

Total RNA from Lbr-6018 and Lbr-35 RNA were extracted from cell pellets in RNA protect at GENEWIZ. Subsequently, ribosomal depletion and cDNA libraries were generated before sequencing on an Illumina HiSeq with paired-end 150 bp technology (Illumina, San Diego, CA, USA). Sequencing reads were checked for quality using FASTQC and mapped to either the Lbr-6108 reference genome, or the Lbr-35 reference genome using Salmon v.1.3.0 [71], orchestrated with GNU parallel v.20161222 [72]. MultiQC was used to summarize quality metrics of read quality and mapping quality, and the median number of reads mapped was 12.3 million (minimum 8.1, maximum 15.8). The graphs and analysis were generated using R v.3.6.3 (Vienna, Austria). Sets of differentially expressed genes were visualized using ComplexHeatmap v2.2.0 [73], and other data visualized using pheatmap v1.0.12 [74] and ggplot2 v3.3.2 [75].

### Statistical analysis

Growth comparisons were analyzed when appropriate using two-way ANOVA with Tukey’s multiple comparisons test in Prism. Values were considered significant their p-value was equal to or less than 0.05. Differentially expressed genes were determined using DESeq2 v1.26.0 [76], with genes called as being significantly differentially expressed if they had an adjusted p-value equal to or less than 0.05 and their absolute value log2 fold change was ± 1.5 or higher.

## Supplementary Information

**Additional file 1: Table S1.** Measurement of glutamate and GABA in *Levilactobacillus brevis* Lbr-6108, Lbr-35, and ATCC 14869. **Table S2.** Glutamate utilization and GABA production by *Levilactobacillus brevis* Lbr-6108 in MRS with different concentrations of MSG. **Table S3.** Gene List from Figure 4. Locus IDs are for *Levilactobacillus brevis* Lbr-6108 genome. **Figure S1.** Culture purity testing by species- and strain-specific PCR for *Levilactobacillus brevis* Lbr-6108 and Lbr-35. **Figure S2.** PCA plot of RNA-sequencing for *Levilactobacillus brevis* Lbr-6108 and Lbr-35.

## Data Availability

The Lbr-6108 genome was published previously and has the National Center for Biotechnology Information (NCBI) accession number MDUA01. Similarly, the genome for ATCC 14869 has the NCBI accession number AWVK01. The novel Lbr-35 genome has accession number JAHERW000000000. The RNA sequencing data are submitted to the NCBI Gene Expression Omnibus (GEO) Database under BioProject accession number PRJNA752280 and GEO accession number GSE181504.

## Abbreviations

APF: Aggregation promoting factor
AQC: Aminoquinolyl-*N*-hydroxysuccinimidyl carbamate
ATCC 14869: *Levilactobacillus brevis* ATCC 14869
bp: Base pair
CNS: Central nervous system
DEG: Differentially expressed gene
ec: Enzyme class
ENS: Enteric nervous system
GABA: Gamma-aminobutyric acid
GAD: Glutamate decarboxylase
Glu: Glutamate
HPLC: High performance liquid chromatography
LAB: Lactic acid bacteria
LC–MS: Liquid chromatography–mass spectrometry
Lbr-6108: *Levilactobacillus brevis* Lbr-6108™
Lbr-35: *Levilactobacillus brevis* Lbr-35™
MRS: De Man-Rogosa-Sharpe broth
MSG: Monosodium glutamate
PLP: Pyridoxal-5′-phosphate
SD: Safe deposit
