# Supplementary material for: Transcriptomics reveal different metabolic strategies for acid resistance and gamma-aminobutyric acid (GABA) production in select Levilactobacillus brevis strains
Source: Microb Cell Fact. 2021 Sep 6;20:173. doi: 10.1186/s12934-021-01658-4 (PMC8419935; doi:10.1186/s12934-021-01658-4)

Table S1. Measurement of glutamate and GABA in *Levilactobacillus brevis* Lbr-6108, Lbr-35, and ATCC 14869. The p-value is the result of an ANOVA with Tukey's multiple comparison test comparing the ODs of each strain to at T48 to Lbr-6108 in the respective media.

		utamate, MRS (µg/ml		GABA, MRS (µg/mL)		OD, MRS (µg/mL)			Glutamate, MRS+MSG (µg/mL)		GABA, MRS+MSG (µg/mL)		OD, MRS+MSG (600nm)		
**Strain**	**Timepoint (hr)**	**rep 1**	**rep 2**	**rep 1**	**rep 2**	**rep 1**	**rep 2**	**p-value**	**rep 1**	**rep 2**	**rep 1**	**rep 2**	**rep 1**	**rep 2**	**p-value**
Lbr-6108	0	660	670	50	50	0.139	0.141		8900	8800	50	50	0.137	0.133	
Lbr-6108	3	650	640	50	50	0.434	0.432		9000	8600	60	58	0.412	0.397	
Lbr-6108	6	620	590	66	70	0.667	0.671		8600	8600	100	100	0.615	0.602	
Lbr-6108	9	570	510	210	190	1.056	1.069		8700	8500	310	320	0.959	0.968	
Lbr-6108	12	360	350	340	350	1.291	1.320		8100	8100	620	630	1.217	1.226	
Lbr-6108	15	130	120	500	520	1.478	1.448		7200	7600	1200	1200	1.406	1.386	
Lbr-6108	18	100	100	500	500	1.638	1.611		6300	6300	1900	2000	1.542	1.540	
Lbr-6108	21	100	100	500	500	1.767	1.763		5000	5100	2800	2800	1.689	1.722	
Lbr-6108	24	100	100	500	500	1.816	1.833		3700	3700	4000	3800	1.789	1.774	
Lbr-6108	48	100	100	500	510	1.733	1.753		250	300	6000	6000	1.724	1.733	
Lbr-35	0	690	670	50	50	0.142	0.136		8900	8800	53	50	0.145	0.139	
Lbr-35	3	680	660	50	50	0.519	0.500		8900	9000	50	50	0.476	0.491	
Lbr-35	6	670	640	50	50	0.855	0.849		8800	8600	50	50	0.780	0.753	
Lbr-35	9	670	700	54	59	1.086	1.110		8600	8900	76	70	1.029	1.021	
Lbr-35	12	710	680	58	50	1.278	1.271		8600	9000	61	56	1.159	1.144	
Lbr-35	15	690	680	50	51	1.446	1.397		8800	8900	75	60	1.266	1.278	
Lbr-35	18	680	690	51	52	1.556	1.544		8700	8900	63	63	1.397	1.401	
Lbr-35	21	670	680	54	58	1.650	1.687		8900	9500	71	72	1.502	1.564	
Lbr-35	24	630	600	82	80	1.741	1.738		8700	8800	120	110	1.566	1.579	
Lbr-35	48	560	540	170	160	1.690	1.690	>0.9999	9000	8900	270	230	1.635	1.621	>0.9999

Table S2. Glutamate utilization and GABA production by *Levilactobacillus brevis* Lbr-6108 in MRS with different concentrations of MSG.

	MRS with 10,000 µg/mL MSG						MRS with 30,000 µg/mL MSG						MRS with 90,000 µg/mL MSG					
	Glu Uptake (µg/mL)		GABA (µg/mL)		**% Yield**		Glu Uptake (µg/mL)		GABA (µg/mL)		**% Yield**		Glu Uptake (µg/mL)		GABA (µg/mL)		% Yield	
**Timepoint (hr)**	**rep 1**	**rep 2**	**rep 1**	**rep 2**	**rep 1**	**rep 2**	**rep 1**	**rep 2**	**rep 1**	**rep 2**	**rep 1**	**rep 2**	**rep 1**	**rep 2**	**rep 1**	**rep 2**	**rep 1**	**rep 2**
5	500.0	450.0	50.0	50.0	**10.0%**	**11.1%**	1000.0	340.0	50.0	50.0	**5.0%**	**14.7%**	5000.0	4740.0	50.0	50.0	1.0%	1.1%
24	4200.0	4290.0	2600.0	2620.0	**61.9%**	**61.1%**	6000.0	5920.0	2900.0	2930.0	**48.3%**	**49.5%**	4000.0	3790.0	1800.0	1830.0	45.0%	48.3%
48	8200.0	8160.0	5300.0	5310.0	**64.6%**	**65.1%**	16300.0	16270.0	10000.0	9970.0	**61.3%**	**61.3%**	24000.0	23530.0	13000.0	12680.0	54.2%	53.9%
72	8000.0	7980.0	6100.0	6075.0	**76.3%**	**76.1%**	18200.0	18405.0	12000.0	12140.0	**65.9%**	**66.0%**	28000.0	23530.0	16000.0	16450.0	57.1%	69.9%

Table S3. Gene List from Figure 4. Locus IDs are for *Levilactobacillus brevis* Lbr-6108 genome. Encoded protein functions are given for genes shown in Figure 4.

**Locus ID**	***Gene name***	**Protein function**	**Operon / pathway**	**Panel in Figure 4**
BGC39_04180	*gadA*	Glutamate decarboxylase (EC 4.1.1.15) GadA	L-Glutamate decarboxylase operon	A
BGC39_07245	*gadR*	Transcriptional regulator GadR	L-Glutamate decarboxylase operon	A
BGC39_07250	*gadC*	Glutamate/gamma-aminobutyrate antiporter	L-Glutamate decarboxylase operon	A
BGC39_07255	*gadB*	Glutamate decarboxylase (EC 4.1.1.15) GadB	L-Glutamate decarboxylase operon	A
BGC39_07260	*gltX*	Glutamyl-tRNA synthetase (EC 6.1.1.17; EC 6.1.1.24)	L-Glutamate decarboxylase operon	A
BGC39_05810	*hmpT*	Substrate-specific component HmpT of predicted hydroxymethylpyrimidine ECF transporter	Pyridoxal 5'-phosphate (PLP)	B
BGC39_05815	*thiD2*	Novel pyridoxal kinase, thiD family (EC 2.7.1.35)	Pyridoxal 5'-phosphate (PLP)	B
BGC39_05820	*norD*	Transcriptional regulator of pyridoxine metabolism / Pyridoxamine phosphate aminotransferase (EC 2.6.1.54)	Pyridoxal 5'-phosphate (PLP)	B
BGC39_05050	*argF*	Ornithine carbamoyltransferase (EC 2.1.3.3)	Arginine deiminase operon	C
BGC39_05055	*arcA*	Arginine deiminase (EC 3.5.3.6)	Arginine deiminase operon	C
BGC39_05060	*arcD*	Arginine/ornithine antiporter ArcD	Arginine deiminase operon	C
BGC39_06170	*aguA*	Agmatine deiminase (EC 3.5.3.12)	Agmatine deiminase operon	D
BGC39_06175	*aguD*	Agmatine/putrescine antiporter, associated with agmatine catabolism	Agmatine deiminase operon	D
BGC39_06180	*aguB*	Putrescine carbamoyltransferase (EC 2.1.3.6)	Agmatine deiminase operon	D
BGC39_06130	*mleP*	Malate permease	Malolactic fermentation operon	E

BGC39_06135	*mleS*	Malolactic enzyme (EC 4.1.1.101)	Malolactic fermentation operon	E
BGC39_06140	*mleR*	Malolactic regulator MleR	Malolactic fermentation operon	E
BGC39_06185	*nhaC2*	Predicted tyrosine transporter, NhaC family	Tyrosine decarboxylase operon	F
BGC39_06190	*tdcP*	Predicted tyrosine transporter, GadC family	Tyrosine decarboxylase operon	F
BGC39_06195	*tdcA*	L-tyrosine decarboxylase (EC 4.1.1.25) TDC	Tyrosine decarboxylase operon	F
BGC39_06200	*tyrS*	Tyrosyl-tRNA synthetase (EC 6.1.1.1)	Tyrosine decarboxylase operon	F

Figure S1. Culture purity testing by species- and strain-specific PCR for *Levilactobacillus brevis* Lbr-6108 and Lbr-35.

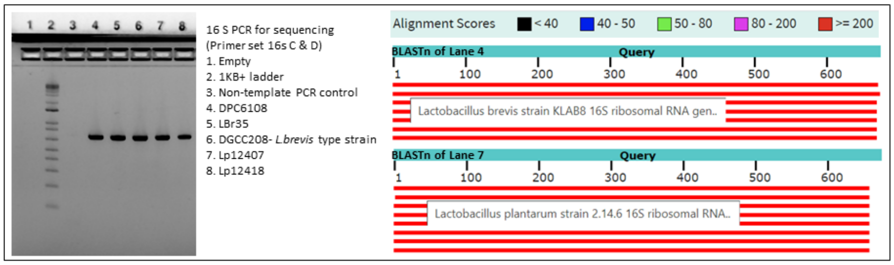

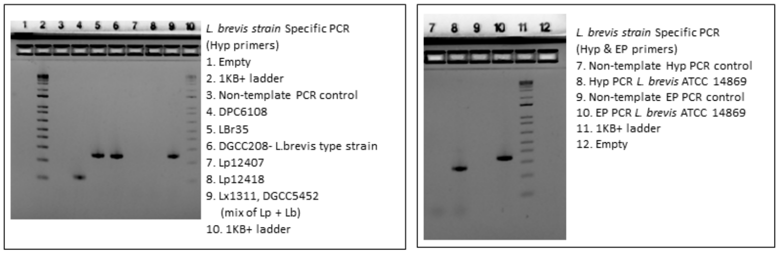

Primer name	Sequence (5'-3')	Length (bp)
Hyp_f	GAACTTCATCAGTAGTGCGTTA	DPC 6108: 130; ATCC 14869 & Lbr-35: 267
Hyp_r	TGTTGGTCTTCGATATAGGTTAG	
EP_f	ACGTCTGGTTATAGCTCATCA	DPC 6018: 213; ATCC 14869 & Lbr-35: 339
EP_r	TAGTTTATCGACCGAGCCTT	
16S C	AACAGGATTAGATACCCTGGTAGTCC	~700
16S D	TACGGCTACCTTGTTACGACTT	

Figure S2. PCA plot of RNA-sequencing for *Levilactobacillus brevis* Lbr-6108 and Lbr-35.

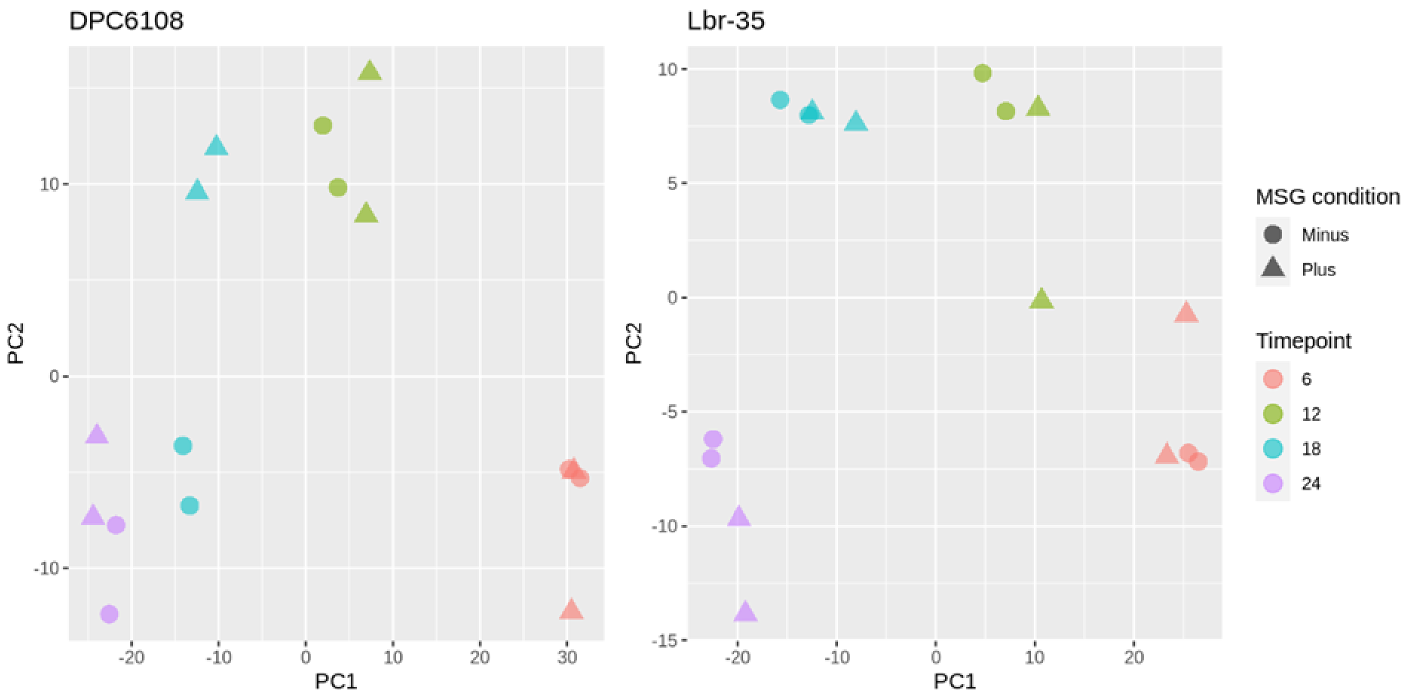

Supplement: Supplementary file 1 — Additional file 1: Table S1. Measurement of glutamate and GABA in Levilactobacillus brevis Lbr-6108, Lbr-35, and ATCC 14869. Table S2. Glutamate utilization and GABA production by Levilactobacillus brevis Lbr-6108 in MRS with different concentrations of MSG. Table S3. Gene List from Figure 4. Locus IDs are for Levilactobacillus brevis Lbr-6108 genome. Figure S1. Culture purity testing by species- and strain-specific PCR for Levilactobacillus brevis Lbr-6108 and Lbr-35. Figure S2. PCA plot of RNA-sequencing for Levilactobacillus brevis Lbr-6108 and Lbr-35. [file 12934_2021_1658_MOESM1_ESM.pdf]
